# CRISPR/Cas9-Mediated Polyketide Synthase Replacement for High-Yield Biosynthesis and Biological Activity of Milbemycin D

**DOI:** 10.3390/biology15070535

**Published:** 2026-03-27

**Authors:** Shenchen Tao, Huan Qi, Xian Luo, Jingyi Shen, Yunfei He, Jun Huang, Ruijun Wang, Shaoyong Zhang, Yongsheng Gao, Jidong Wang, Liqin Zhang

**Affiliations:** 1 Collaborative Innovation Center of Green Pesticide, School of Forestry and Biotechnology, Zhejiang A & F University, No. 666 Wusu Street, Linan District, Hangzhou 311300, China; shenchentaoh@163.com; 2 Zhejiang Key Laboratory of Biology and Ecological Regulation of Crop Pathogens and Insects, College Life Science, Huzhou University, No. 759 Erhuandong Road, Wuxing District, Huzhou 313000, China; 02765@zjhu.edu.cn (H.Q.); 19857220376@163.com (X.L.); 18267253399@163.com (J.S.); iheyunfei@163.com (Y.H.); hachael@126.com (J.H.); 02753@zjhu.edu.cn (R.W.); 02703@zjhu.edu.cn (S.Z.); gaoys@zjhu.edu.cn (Y.G.); 02752@zjhu.edu.cn (J.W.)

**Keywords:** milbemycins, avermectins, CRISPR/Cas9, genome editing, polyketide synthase, combinatorial biosynthesis, biological activity

## Abstract

Milbemycin D is a promising 16-membered macrolide insecticide with reported superior efficacy, but its commercial development has been hindered by extremely low natural yields. This study aimed to construct a high-yielding microbial platform for milbemycin D production using combinatorial biosynthesis and advanced genome editing. An optimized CRISPR/Cas9-AcrIIA4 system was employed to seamlessly replace the *aveA3* polyketide synthase (PKS) gene in the ivermectin B1b-producing strain *Streptomyces avermitilis* HU501 with the heterologous *milA3* PKS from *S. bingchenggensis*. The engineered strain was validated genetically and metabolically, followed by high-throughput screening and fermentation optimization in various media. The biosynthesized compound was structurally confirmed by spectroscopy. Bioactivity was evaluated against *Bursaphelenchus xylophilus*, *Hyphantria cunea*, and *Plutella xylostella*. The engineered strain *S. avermitilis* HU501-M successfully shifted its major product to milbemycin D, reaching a final titer of 679.03 mg/L. Bioassays revealed that milbemycin D exhibited significantly enhanced potency, with LC_50_ values 8–24% lower than those of milbemycin A3/A4. This work demonstrates an efficient CRISPR/Cas9-mediated PKS replacement strategy to achieve the high-yield production of milbemycin D, offering a promising microbial source and a generalizable framework for engineering complex polyketide pathways. This proof-of-concept establishes a foundation for future process development toward potential commercial application.

## Simple Summary

This study addresses the low natural yield of milbemycin D, a potent but underutilized insecticidal macrolide, by engineering a high-producing microbial strain. Using an optimized CRISPR/Cas9-AcrIIA4 system, we replaced the *aveA3* polyketide synthase gene in *Streptomyces avermitilis* HU501 with the heterologous *milA3* gene from *Streptomyces bingchenggensis*, effectively redirecting biosynthesis toward milbemycin D. Through fermentation optimization, a titer of 679.03 mg/L was achieved. Bioassays demonstrated that the biosynthesized milbemycin D exhibits enhanced activity against key pests compared to commercial milbemycin A3/A4. This work establishes a high-yield, laboratory-scale platform for milbemycin D production and highlights CRISPR/Cas9-driven combinatorial biosynthesis as a powerful tool for accessing high-value natural products.

## 1. Introduction

**Figure 1:**
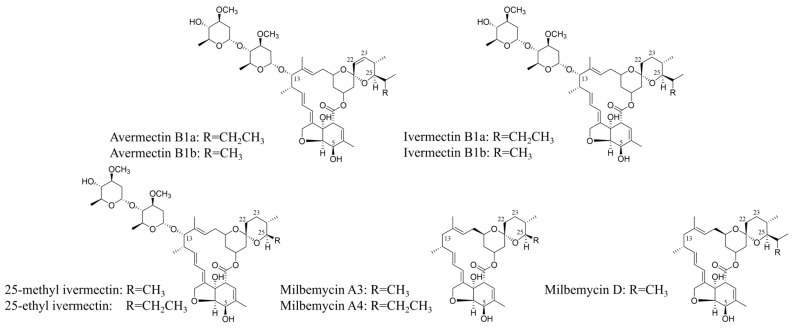
The structures of avermectins and milbemycins.

Milbemycins are a family of 16-membered macrolides structurally related to the avermectins, renowned for their potent anthelmintic and insecticidal activities [1]. The core structural distinctions between milbemycins and avermectins reside at three key positions: the substituent at C25, the saturation state of the C22–C23 bond, and the presence or absence of a β-L-oleandrosyl disaccharide at C13 (Figure 1) [2]. Through chemical modification of these sites, a series of commercially successful derivatives have been developed, including ivermectin, abamectin, doramectin, and milbemycin oxime, which are cornerstone agents in veterinary and agricultural pest management [2]. Among these, milbemectin—a mixture of approximately 30% milbemycin A3 and 70% milbemycin A4—exemplifies the desirable attributes of this class: broad-spectrum efficacy, high potency, and selective toxicity that spares beneficial organisms and mammals [3,4,5].

Milbemycin D, a natural congener produced as a minor component by *Streptomyces hygroscopicus*, possesses a unique isopropyl group at C25, distinguishing it from the methyl (A3) and ethyl (A4) substituents of its counterparts [6]. Notably, milbemycin D has demonstrated superior and more persistent efficacy against parasites such as *Dirofilaria immitis* compared to milbemycins A3 and A4 at equivalent doses, highlighting its significant potential [7]. However, its commercial development has been severely hampered by its extremely low yield in native fermentations, which results in prohibitively high production costs and leaves its full insecticidal spectrum poorly characterized [8,9].

**Figure 2:**
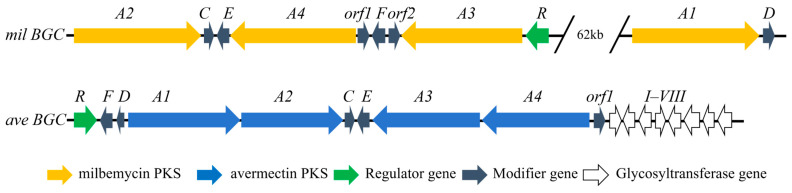
Gene of milbemycin (mil) and avermectin (ave) biosynthetic gene clusters.

The biosynthetic gene clusters (BGCs) for milbemycins in *Streptomyces nanchangensis*, *Streptomyces bingchenggensis*, and for avermectins in *Streptomyces avermitilis* have been elucidated [10,11,12]. The milbemycin BGC typically comprises four large type-I polyketide synthase (PKS) genes (*milA1*–*A4*), alongside tailoring and regulatory genes [11]. A notable feature is the discontinuous organization of its PKS genes compared to the contiguous avermectin PKS (Figure 2). This genomic architecture, among other factors, contributes to the generally lower fermentation titers of milbemycins versus avermectins, presenting an economic barrier to their wider application [13]. Consequently, strain improvement is critical. While strategies like regulator overexpression (*milR*) [14], targeted gene inactivation (*milD*, *cyp41*) [15,16], and manipulation of global regulators (*mtrA_sbh_*) [17] have successfully boosted milbemycin A3/A4 yields, effective approaches to enhance the production of the more potent milbemycin D remain lacking.

Combinatorial biosynthesis, particularly the replacement of PKS modules, offers a powerful strategy to redirect metabolic flux or generate novel compounds. For instance, substituting domains within the avermectin PKS with counterparts from oligomycin or milbemycin PKS have yielded ivermectin-producing strains and novel avermectin derivatives [18,19]. The critical enabler of such strategies is an efficient genome editing tool. Traditional methods in *Streptomyces*, relying on suicide plasmids and double-crossover recombination, are notoriously inefficient and time-consuming [20]. While improved systems like PCR-targeting, Cre-*lox*P, and meganuclease (I-SceI) recombination have been developed, they often leave genetic scars or require pre-engineered recognition sites, limiting their versatility for complex engineering [21,22,23,24,25].

The advent of CRISPR/Cas9 technology has revolutionized genetic manipulation in *Streptomyces* by enabling precise, targeted double-strand breaks via programmable sgRNAs, without the need for pre-modified genomes [26,27]. Initial challenges, such as Cas9 toxicity and off-target effects, have been mitigated through innovations like inducible promoters, split-Cas9 systems, ATP synthase overexpression, and the use of anti-CRISPR proteins (e.g., AcrIIA4), leading to highly optimized and efficient editing platforms [28,29,30,31,32].

In this study, we aimed to address the critical production bottleneck of milbemycin D by employing a combinatorial biosynthesis strategy. We leveraged an optimized CRISPR/Cas9-AcrIIA4 system to engineer the industrially relevant chassis strain *S. avermitilis* HU501. Our objective was to replace the native *aveA3* PKS module with the heterologous *milA3* module from *S. bingchenggensis*, thereby redirecting biosynthesis to establish milbemycin D as the primary fermentation product. This work not only seeks to develop a viable microbial platform for the efficient production of this high-potential compound but also aims to demonstrate a refined and efficient method for large-scale, seamless PKS module replacement in *Streptomyces*. The successful implementation of this strategy would provide a generalizable framework for the combinatorial biosynthesis of complex polyketides and facilitate the industrial development of promising but previously inaccessible natural product derivatives.

## 2. Materials and Methods

### 2.1. Bacterial Strains, Plasmids, and Culture Conditions

All bacterial strains and plasmids used in this study are listed in Table 1. The parental strain *Streptomyces avermitilis* AVE-H39 was maintained on YMS agar (per liter: 4 g yeast extract, 4 g soluble starch, 10 g malt extract, 20 g agar) [20]. To generate a suitable chassis strain for this work, *S. avermitilis* AVE-H39 was subjected to sequential rounds of mutagenesis using ultraviolet (UV) irradiation, atmospheric and room temperature plasma (ARTP), and ethyl methanesulfonate (EMS) [33].

Spore preparation: Spores of *S. avermitilis* AVE-H39 were cultivated on ISP2 or ISP3 agar at 28 °C for 8–10 days. Under aseptic conditions, spores were scraped and suspended in sterile water to a concentration of 10^7^ spores/mL.

UV mutagenesis: 1 mL of spore suspension was spread onto a sterile 9-cm Petri dish and irradiated with a 30 W UV lamp (λ = 254 nm) at a distance of 20 cm for 40, 60, or 80 s. Following red-light illumination, the suspension was serially diluted to 10^−4^, 10^−5^, and 10^−6^ in physiological saline, plated on ISP2 agar, and incubated in darkness at 28 °C for 8 days. Surviving colonies were isolated.

ARTP mutagenesis: 25 μL of spore suspension was applied to a sample carrier slide and treated using an ARTP mutagenesis system with a gas flow rate of 10 SLM, radiofrequency power input of 100 W, and sample distance of 2 mm for 30, 40, 50, or 60 s. Treated suspensions were serially diluted to 10^−4^, 10^−5^, and 10^−6^ in sterile water, plated on ISP2 agar, and incubated at 28 °C for 8 days. Mutant colonies were selected.

EMS mutagenesis: EMS solution (4.0% *v*/*v*) was prepared by dissolving 1 mL EMS in 2 mL absolute ethanol, then adding to 22 mL phosphate buffer (pH 7.2). For treatment, 2 mL of spore suspension was mixed with EMS solution to final concentrations of 2.0%, 3.0%, or 4.0% (*v*/*v*) and shaken at 150 rpm for 30 min. The reaction was quenched with 10 mL of 5% sodium thiosulfate. The suspension was serially diluted to 10^−4^, 10^−5^, and 10^−6^ in physiological saline, plated on ISP2 agar, and incubated at 28 °C for 8 days. Mutant colonies were selected.

Combined mutagenesis: Sequential treatments using two or more of the above methods were applied to spore suspensions.

Screening procedure: A minimum of 100 single colonies from each mutagenesis round were selected and cultivated individually on ISP2 agar. Spores were harvested for shake-flask fermentation in original medium (OG) containing (per liter): corn starch 100 g, amylase 0.2 g, glucose 10 g, yeast powder 10 g, soybean cake powder 20 g, CaCO_3_ 3 g, pH 7.0 [34]. Fermentation was conducted in 250-mL flasks containing 30 mL medium at 28 °C and 250 rpm for 10 days.

For HPLC analysis, 1 mL of fermentation broth was mixed with 3 mL methanol, sonicated for 30 min, and filtered. The filtrate was analyzed using an Agilent 1260 Infinity II system (Agilent, Santa Clara, CA, USA) with a ZORBAX XDB-C18 column (4.6 mm × 250 mm, 5 μm). The mobile phase was methanol:acetonitrile:water = 81:7:12 (*v*/*v*/*v*) at 1.0 mL/min, with detection at 240 nm. Strains producing ivermectin B1b (C_47_H_72_O_14_) were identified by comparison with an authentic standard.

After multiple rounds of mutagenesis and screening, the mutant strain *S. avermitilis* HU501 was obtained, exhibiting stable ivermectin B1b production. Based on product analysis, we infer that the mutagenesis introduced genetic changes that altered the selectivity of starter units, shifting incorporation from acetyl-CoA and propionyl-CoA (used for 25-methyl and 25-ethyl ivermectin) to isobutyryl-CoA (used for ivermectin B1b), although the precise mutations have not been characterized at the sequence level. This strain has been deposited at the China Center for Type Culture Collection under accession number CCTCC M 2025824.

For genetic manipulation, the temperature-sensitive *E. coli*-*Streptomyces* shuttle vector pKC1139-Cas9, which harbors a fine-tuned expression system for high Cas9 activity, was used as the base plasmid [32]. To minimize off-target effects, four distinct sgRNAs were designed in silico against unique regions of the *aveA3* sequence using the online tool at https://autoesdcas.biodesign.ac.cn (accessed on 21 September 2024). The spacer with the highest predicted on-target efficiency and the fewest potential off-target sites within the *S. avermitilis* HU501 genome was selected for plasmid construction. The complete *milA3* polyketide synthase (PKS) gene sequence was sourced from the genome of *S. bingchenggensis* BCW-1 (GenBank accession GCF_000092385.1) [11]. This gene, along with all primers (Table S1), was commercially synthesized in segmented form (Sangon Biotech, Shanghai, China). Standard molecular biology techniques were employed for genomic DNA isolation, PCR amplification using GXL DNA polymerase (Takara, Kusatsu, Shiga, Japan), and cloning [20,35]. Assembly of the final replacement plasmid, pKC1139-Cas9-spacer-AA3UD-MA3, was achieved using restriction endonucleases (Thermo Fisher Scientific, Waltham, Massachusetts, USA) and a One Step Cloning Kit (Vazyme, Nanjing, Jiangsu, China). Constructs were propagated in *E. coli* DH5α cultured in LB medium supplemented with apramycin (50 µg/mL) [35]. All plasmid assemblies were verified by single-molecule sequencing (Sangon Biotech). For conjugal transfer, recombinant plasmids were introduced into the non-methylating donor strain *E. coli* ET12567/pUZ8002 and transferred to *S. avermitilis* HU501 on solid mannitol-soybean (MS) medium as described previously [20].

### 2.2. Construction of the aveA3 Replacement Mutant Strain S. avermitilis HU501-M

To engineer the *aveA3* replacement mutant, the recombinant plasmid pKC1139-Cas9-spacer-AA3UD-MA3 was constructed for CRISPR/Cas9-mediated homologous recombination. Using the pKC1139-Cas9 plasmid as a template, a 208-bp fragment containing the *aveA3*-targeting sgRNA (spacer) sequence was amplified with primers T1 and T2. Concurrently, a 141-bp fragment was amplified from the same template using primers T3 and T4. These two fragments served as templates for a fusion PCR with primers T2 and T3, yielding a final 329-bp fragment. The pKC1139-Cas9 plasmid was digested with XbaI/NheI and ligated with the sgRNA-containing fragment using a One Step Cloning Kit to generate pKC1139-Cas9-spacer.

Next, homology arms flanking the *aveA3* locus were amplified from *S. avermitilis* HU501 genomic DNA: a 2.7-kb upstream fragment (primers T5 and T6) and a 3.0-kb downstream fragment (primers T7 and T8). These fragments were inserted into the EcoRI site of pKC1139-Cas9-spacer using seamless assembly, yielding pKC1139-Cas9-spacer-AA3UD. An NdeI restriction site was introduced between the homology arms to facilitate subsequent insertion of the *milA3* sequence.

Due to its large size (~17.4 kb) and high GC content, the full-length *milA3* PKS gene was synthesized as four overlapping fragments of approximately 4.6 kb, 3.6 kb, 4.1 kb, and 5.1 kb. Although simultaneous ligation of all four fragments was possible, efficiency was low. Therefore, a sequential strategy was employed. First, the 4.6-kb (fragment 1) and 5.1-kb (fragment 4) segments were ligated into the NdeI site of pKC1139-Cas9-spacer-AA3UD, resulting in the intermediate plasmid pKC1139-Cas9-spacer-AA3UD-MA3part. Because the seamless assembly obscured the original NdeI site, a new NdeI site was reintroduced between the inserted fragments. Finally, the remaining 3.6-kb (fragment 2) and 4.1-kb (fragment 3) segments were ligated into this site, completing the assembly of the full-length milA3 gene and yielding the final replacement plasmid pKC1139-Cas9-spacer-AA3UD-MA3. To ensure the fidelity of this large construct, the entire assembled plasmid was subjected to full-length sequencing after each sequential cloning step. The resulting sequences were aligned against the expected reference using ClustalW (https://www.genome.jp/tools-bin/clustalw, accessed on 15 November 2024), and only plasmids exhibiting 100% concordance were advanced to the next stage of construction or used for conjugation.

The plasmid was verified by PCR and restriction digestion, then transformed into the non-methylating E. coli donor strain ET12567/pUZ8002 [20]. Conjugation with *S. avermitilis* HU501 was performed on solid MS agar (2% mannitol, 2% soybean cake powder, 2% agar). After 2 days of incubation at 30 °C, the plates were overlaid with apramycin (50 µg/mL) and nalidixic acid (25 µg/mL) to select for exconjugants. Apramycin-resistant colonies were allowed to develop for 5–7 days at 30 °C, then transferred to YMS agar with apramycin for amplification.

To cure the temperature-sensitive plasmid, colonies were inoculated onto non-selective YMS plates and incubated at 37 °C for 24 h, then returned to 30 °C for 5–7 days. Candidate mutants were screened by PCR using primers milA3check-F and milA3check-R (Table S1). Approximately 500 initial exconjugants were screened; after plasmid curing, approximately 300 clones were confirmed by PCR to have stably integrated the milA3 gene. A clone showing the expected 3.1-kb milA3 amplicon was selected, preserved in 20% glycerol, and designated *S. avermitilis* HU501-M.

### 2.3. Fermentation and HPLC Analysis of S. avermitilis HU501-M

The engineered strain was cultivated in seed medium containing corn starch (20 g/L), glucose (5 g/L), yeast extract (10 g/L), soybean cake powder (10 g/L), and CaCO_3_ (2 g/L), pH 7.0. Cultures were incubated at 28 °C with shaking at 200 rpm for 40–48 h. Then, 2 mL of seed culture was inoculated into 250 mL Erlenmeyer flasks containing 30 mL of the OG medium. Fermentation proceeded at 28 °C and 220 rpm on a Zhicheng constant temperature culture oscillator (model ZWYR-2112D, Zhicheng, Shanghai, China) for 180–240 h.

For HPLC analysis, 2 mL of fermentation broth was mixed with 6 mL of ethanol, vortexed vigorously, and sonicated for 30 min. The supernatant was filtered through a 0.22 µm membrane and analyzed using an Agilent 1260 Infinity II system equipped with a Supersil ODS2 C_18_ column (4.6 mm × 200 mm, 5 µm). The mobile phase consisted of CH_3_CN/CH_3_OH/H_2_O (65:23:12, *v*/*v*/*v*) run at a flow rate of 1.0 mL/min, with detection at 240 nm.

### 2.4. Purification of Milbemycin D

For large-scale purification, 15 L of fermentation broth was centrifuged at 4500 rpm for 15 min. The cell pellet was washed with 5 L of methanol, sonicated for 30 min, and centrifuged again. The methanol extract was concentrated under reduced pressure at 50 °C to approximately 1 L, then extracted three times with an equal volume of ethyl acetate. The combined organic phases were concentrated to yield approximately 25 g of an oily residue.

This residue was subjected to silica gel column chromatography and eluted with a stepwise gradient of petroleum ether–ethyl acetate (95:5 to 60:40, *v*/*v*). Fractions eluted with 80:20 to 70:30 petroleum ether–ethyl acetate were pooled and concentrated to obtain a crude mixture. Final purification was achieved by semi-preparative HPLC (Agilent 1100 system, Zorbax XDB-C_18_ column, 5 µm, 9.4 mm × 250 mm) using methanol–water (95:5, *v*/*v*) as the mobile phase at 1.5 mL/min, with detection at 240 nm, yielding pure milbemycin D. The purity of the final compound was determined to be 97.715% by HPLC peak area integration at 240 nm (Figure S1). This was confirmed by ^1^H NMR analysis, which showed no detectable major impurities (Figure S2).

### 2.5. Structural Analysis

High-resolution electrospray ionization mass spectrometry (HRESI-MS) was performed on an Agilent 6545 Q-TOF LC/MS spectrometer coupled with an Agilent 1290 HPLC system. The system was equipped with an EclipsePlus C_18_ column (2.1 mm × 50 mm, 1.8 µm). Analysis used a gradient elution with mobile phase A (0.1% formic acid in water) and B (acetonitrile) at a flow rate of 0.3 mL/min, with detection at 254 nm. The ESI source parameters were set to 3.5 kV spray voltage, 325 °C capillary temperature, and a gas flow of 10 L/min.

Nuclear magnetic resonance (NMR) spectra, including ^1^H, ^13^C, and 2D experiments (COSY, HSQC, HMBC), were recorded on a Zhongke Oxford AS400 (^1^H 400 MHz; ^13^C 100 MHz) (Zhongke Oxford, Wuhan, Hubei, China). Samples were dissolved in CDCl_3_ and analyzed using 2.5 mm microcells (Synthware, Tampa, FL, USA). Chemical shifts were referenced to the residual solvent peaks of CDCl_3_ at *δ*_H_ 7.26 ppm and *δ*_C_ 77.23 ppm.

### 2.6. Fermentation Media Composition for Screening

Twelve different fermentation media, adapted from the literature for macrolide production, were screened for milbemycin D yield optimization [14,34,36,37,38,39,40,41,42,43,44,45]. Their compositions were as follows:

(1) OG (Original): Corn starch 100 g/L, amylase 0.2 g/L, glucose 10 g/L, yeast powder 10 g/L, soybean cake powder 20 g/L, CaCO_3_ 3 g/L, pH 7.0.
(2) Avm (Avermectin): Soluble starch 70 g/L, yeast extract 16 g/L, K_2_HPO_4_·3H_2_O 0.5 g/L, MgSO_4_·7H_2_O 0.5 g/L, KCl 4 g/L, CoCl_2_·6H_2_O 0.01 g/L, CaCO_3_ 2 g/L.
(3) Mil (Milbemycin): Sucrose 80 g/L, soybean cake powder 20 g/L, CaCO_3_ 2 g/L, K_2_HPO_4_·3H_2_O 1 g/L, FeSO_4_·7H_2_O 0.1 g/L, pH 7.0.
(4) Dor (Doramectin): Soluble starch 90 g/L, soybean cake powder 15 g/L, cottonseed cake powder 15 g/L, yeast extract 5 g/L, NaCl 1 g/L, K_2_HPO_4_·3H_2_O 2.5 g/L, CaCO_3_ 7 g/L, MgSO_4_·7H_2_O 5 g/L, pH 7.0–7.2.
(5) Nem (Nemadectin): Corn starch 90 g/L, amylase 0.09 g/L, glucose 20 g/L, soybean cake powder 25 g/L, yeast extract 5 g/L, CaCO_3_ 4 g/L, MgSO_4_·7H_2_O 5 g/L, CuSO_4_ 0.01 g/L, CoCl_2_ 0.002 g/L, MnSO_4_ 0.001 g/L, pH 7.4.
(6) Blm (Bleomycin): Soluble starch 10 g/L, glucose 10 g/L, peptone 2.5 g/L, yeast extract 2.5 g/L, CuSO_4_·5H_2_O 0.05 g/L, ZnSO_4_·7H_2_O 0.05 g/L, NaCl 3 g/L, CaCO_3_ 3 g/L, pH 7.0.
(7) Asm (Ascomycin): Soluble starch 20 g/L, maltodextrin 40 g/L, yeast powder 5 g/L, peptone 5 g/L, corn pulp powder 5 g/L, K_2_HPO_4_ 1 g/L, (NH_4_)_2_SO_4_ 1.5 g/L, MnSO_4_ 0.05 g/L, MgSO_4_·7H_2_O 1 g/L, CaCO_3_ 1 g/L, soybean oil 2.5 mL/L.
(8) Rap (Rapamycin): Maltodextrin 20 g/L, soybean cake powder 30 g/L, (NH_4_)_2_SO_4_ 1 g/L, KH_2_PO_4_ 5 g/L, pH 6.8–7.0.
(9) Spi (Spiramycin): Maltodextrin 65 g/L, soybean cake powder 5 g/L, corn pulp powder 3 g/L, (NH_4_)_2_SO_4_ 4 g/L, NaCl 5 g/L, KH_2_PO_4_ 4 g/L, MgSO_4_·7H_2_O 1 g/L, ZnSO_4_·7H_2_O 0.1 g/L, CoCl_2_·6H_2_O 0.003 g/L, CaCO_3_ 10 g/L, pH 7.0.
(10) Tet (Tetramycin): Corn starch 10 g/L, soluble starch 20 g/L, soybean cake powder 10 g/L, KH_2_PO_4_ 3 g/L, NaCl 3 g/L, NH_4_Cl 4 g/L, CaCO_3_ 4 g/L, pH 7.2.
(11) AmB (Amphotericin B): Glucose 69 g/L, cottonseed cake powder 25 g/L, CaCO_3_ 10 g/L, KH_2_PO_4_ 0.1 g/L.
(12) Nat (Natamycin): Glucose 60 g/L, peptone 20 g/L, yeast extract 10 g/L, NaCl 5 g/L, MgSO_4_·7H_2_O 5 g/L, pH 7.2.

Each production medium had four replicates.

### 2.7. Biological Activity Assay

A stock solution of milbemycin D was prepared by dissolving 100 mg in 5 mL of DMSO, then serially diluted to working concentrations of 20, 50, 100, 250, and 500 mg/L. Milbemycin A3 and A4 were prepared at identical concentrations for comparison. A blank control (DMSO in water) was included.

*Bursaphelenchus xylophilus* (Pinewood Nematode) Bioassay: For each concentration, 100 µL of test solution was added to 900 µL of an aqueous suspension containing approximately 2500 live nematodes (third-instar larvae) per mL, resulting in approximately 2250–2750 nematodes per replicate. The mixture was kept at 25 °C for 24 h. Nematodes were considered dead if no movement was observed upon probing with a needle. Mortality was assessed under a microscope. Each concentration and the control were tested in three independent biological replicates (separate nematode batches and freshly prepared solutions).

*Hyphantria cunea* (Fall Webworm) Bioassay: Fourth-instar larvae, starved for 12 h, were used as test organisms. Solutions of milbemycin A3, A4, and D at 2, 5, 10, 25, and 50 mg/L were sprayed evenly onto 7 cm × 7 cm *Morus alba* (mulberry) leaf sections. After air-drying, 15 larvae were used per concentration, with one larva placed on each treated leaf section. Larvae were then transferred to fresh, untreated leaves for an additional 24 h before final mortality assessment. Control leaves were treated with solvent only. Each treatment had three independent biological replicates (separate batches of larvae and freshly prepared solutions).

*Plutella xylostella* (Diamondback Moth) Bioassay: Third-instar larvae, starved for 4 h, were used. Leaves of *Brassica oleracea* were dipped in test solutions of milbemycin A3, A4, and D at concentrations of 0.0625, 0.125, 0.25, 0.5, and 1 mg/L. After drying, 30 larvae were used per concentration, with one larva placed on each treated leaf disc at 25 °C. Mortality was recorded after 48 h. Controls received solvent-treated leaves. All tests were performed in three independent biological replicates.

### 2.8. Data Analysis

IBM SPSS Statistics (Version 18.0) was utilized for one-way analysis of variance (ANOVA) with the significance level set at *p* < 0.05. For all bioassays, dose–response data were analyzed by probit analysis to calculate the median lethal concentration (LC_50_) and corresponding 95% confidence intervals for each compound. Following standard ecotoxicological practice, LC_50_ values were considered significantly different when their 95% confidence intervals did not overlap.

## 3. Results

### 3.1. Construction and Genetic Validation of the milA3 Replacement Strain

**Figure 3:**
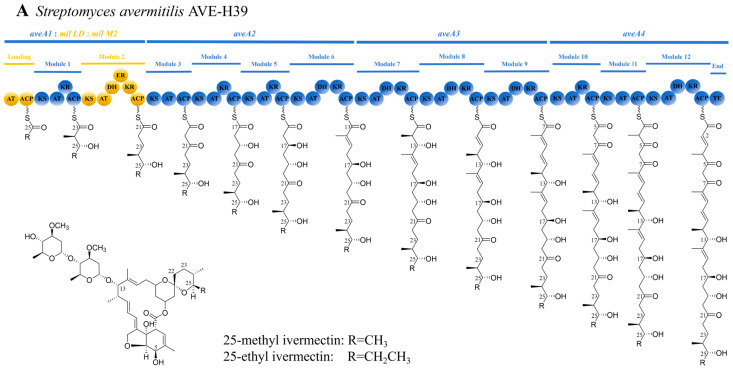

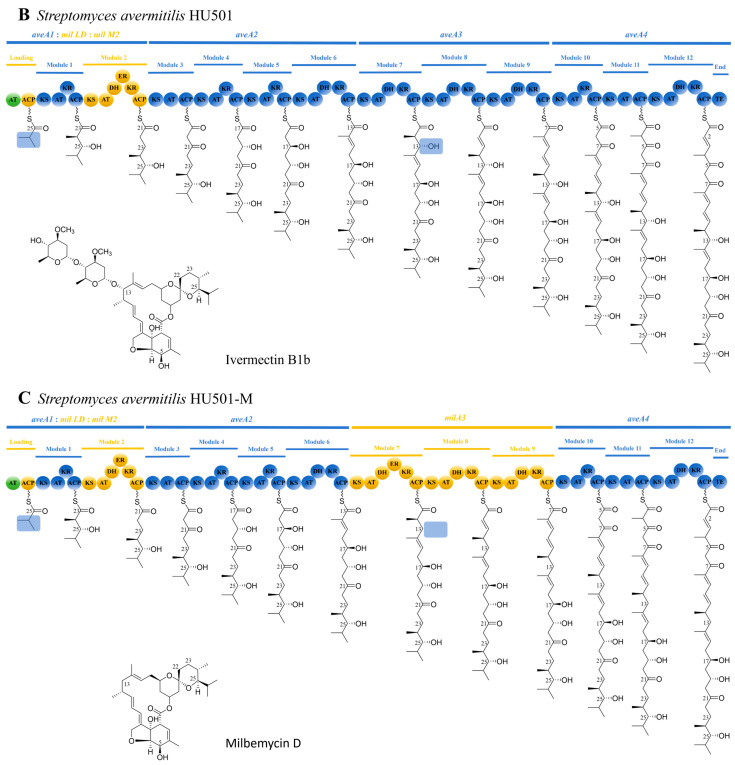
Strategy for engineering milbemycin D biosynthesis via PKS module replacement. Modular organization of the relevant PKS genes in (**A**) the 25-methyl and 25-ethyl ivermectin-producing *S. avermitilis* AVE-H39, (**B**) the mutagenized ivermectin B1b-producing chassis strain *S. avermitilis* HU501, following the mutation in AT domain of Loading PKS, exhibits an altered substrate specificity, and (**C**) the engineered milbemycin D-producing strain *S. avermitilis* HU501-M, following replacement of *aveA3* with *milA3*. The blue areas indicate that the gene sequences are from *Streptomyces avermitilis*. The yellow areas indicate that the gene sequences are from *Streptomyces bingchenggensis*. The green circle indicates that there is a mutation in AT domain. The light blue boxes indicate the key genetic differences that determine product outcome.

**Figure 4:**
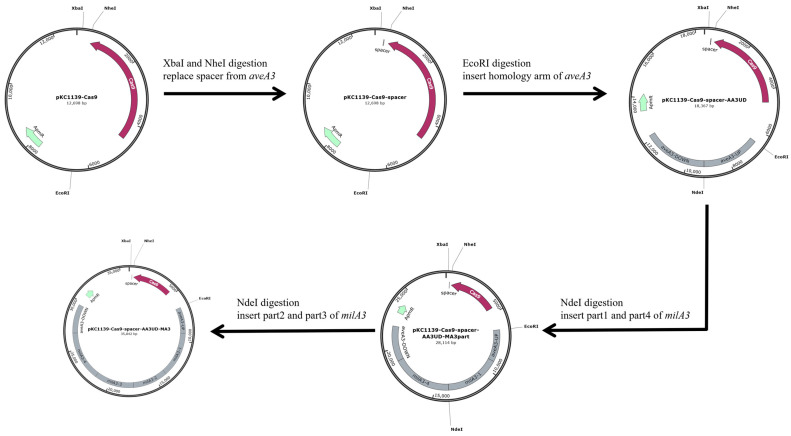
Schematic for constructing the *aveA3* replacement plasmid, pKC1139-Cas9-spacer-AA3UD-MA3. The workflow illustrates the sequential cloning steps: insertion of the sgRNA targeting *aveA3*, addition of homologous flanking arms, and assembly of the full-length *milA3* PKS gene via seamless ligation.

To execute the designed PKS module swap (Figure 3), we employed an optimized CRISPR/Cas9-AcrIIA4 system to replace the *aveA3* gene in *S. avermitilis* HU501 with the heterologous *milA3* from *S. bingchenggensis* BCW-1. We constructed the replacement plasmid pKC1139-Cas9-spacer-AA3UD-MA3 through a sequential cloning strategy (Figure 4). This involved inserting a target-specific sgRNA, flanking homology arms for the *aveA3* locus, and the full-length *milA3* PKS gene, which was synthesized based on the *S. bingchenggensis* sequence and assembled in vitro. Single-molecule sequencing confirmed the seamless assembly and fidelity of the construct.

**Figure 5:**
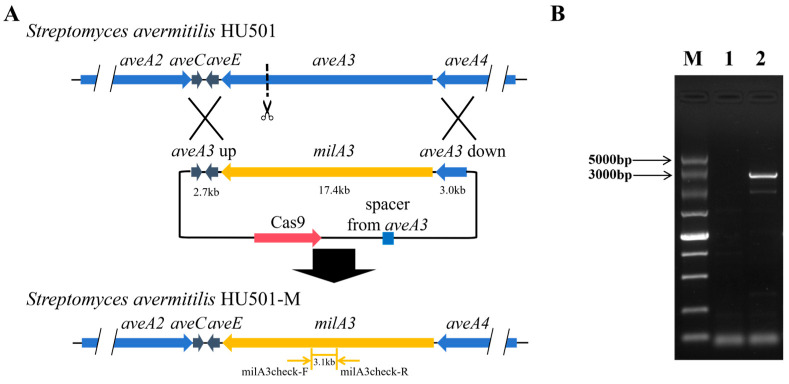
Validation of *milA3* integration into the *S. avermitilis* HU501 genome. (**A**) Schematic of the CRISPR/Cas9-mediated homologous recombination strategy for replacing *aveA3* with *milA3*. (**B**) PCR verification using primers milA3check-F/R. Genomic DNA templates: Lane M, DNA ladder; Lane 1, wild-type *S. avermitilis* HU501; Lane 2, engineered *S. avermitilis* HU501-M. The expected 3.1-kb *milA3* fragment is present only in the engineered strain.

The resulting plasmid was introduced into *S. avermitilis* HU501 via intergeneric conjugation, utilizing a CRISPR/Cas9-mediated homologous recombination strategy designed to replace *aveA3* with *milA3* (Figure 5A). Following conjugation, exconjugants were selected, and the temperature-sensitive vector was cured to obtain plasmid-free candidates. PCR analysis of genomic DNA from these candidates using *milA3*-specific primers (milA3check-F/R) provided genetic confirmation. The expected 3.1-kb amplicon corresponding to the integrated *milA3* sequence was detected exclusively in the engineered strain (designated *S. avermitilis* HU501-M) and was absent in the parental HU501 strain (Figure 5B and Figure S2). This result confirmed the successful and precise replacement of the *aveA3* PKS module within the avermectin biosynthetic gene cluster.

### 3.2. Production and Verification of Milbemycin D by Engineered S. avermitilis HU501-M

**Figure 6:**
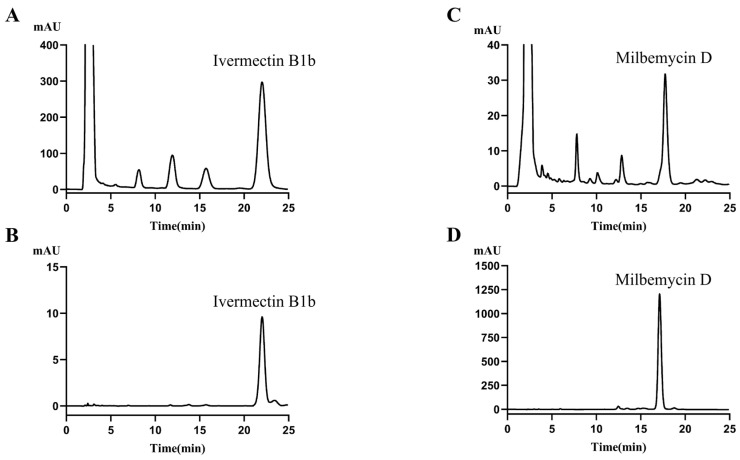
HPLC analysis confirming the metabolic shift from ivermectin B1b to milbemycin D. Chromatograms of fermentation broths from the parent and engineered strains alongside analytical standards: (**A**) *S. avermitilis* HU501, (**B**) ivermectin B1b standard, and (**C**) *S. avermitilis* HU501-M (**D**) milbemycin D standard. Key product peaks are indicated.

Following genetic validation, we characterized the metabolic output of the engineered strain. Initial analysis of the parent chassis, *S. avermitilis* HU501 (derived from AVE-H39 through the sequential mutagenesis and screening described in Section 2.1), by HPLC confirmed ivermectin B1b as its predominant fermentation product, co-eluting with an authentic standard (Figure 6A,B). This confirms that the mutagenesis successfully shifted the starter unit selectivity while retaining the pre-engineered C22–C23 saturation from the AVE-H39 background (Supplementary Table S2 for comparative production data).

Fermentation of the genetically verified *S. avermitilis* HU501-M strain under identical conditions revealed a complete metabolic shift. HPLC analysis of the mycelial extracts showed a new, dominant peak with a retention time of 17.44 min (Figure 6C). This peak co-eluted precisely with an authentic milbemycin D standard (Figure 6D), providing initial chromatographic evidence for the successful biosynthesis of the target compound.

### 3.3. Structural Confirmation of Biosynthesized Milbemycin D

The major compound produced by *S. avermitilis* HU501-M was isolated and purified for structural elucidation. The obtained white amorphous powder was confirmed to be milbemycin D through comprehensive spectroscopic analysis. High-resolution ESI-MS established a molecular formula of C_33_H_48_O_7_ (observed [M + Na]^+^ *m*/*z* 579.3304, calcd. 579.3306) (Figure S3). Comparison of its ^1^H and ^13^C NMR data (Table S3) with the literature values confirmed the milbemycin scaffold [46]. The key distinguishing feature—an isopropyl group at C25—was evident from two characteristic methyl doublets (*δ*H 0.88 and 1.05, each 3H), differentiating it from the methyl (milbemycin A3) or ethyl (milbemycin A4) substituents at this position. Full 1D and 2D NMR spectra are provided in the Supplementary Materials (Figures S3–S6; Table S3).

### 3.4. Screening of the High-Yielding Milbemycin D Strain

**Figure 7:**
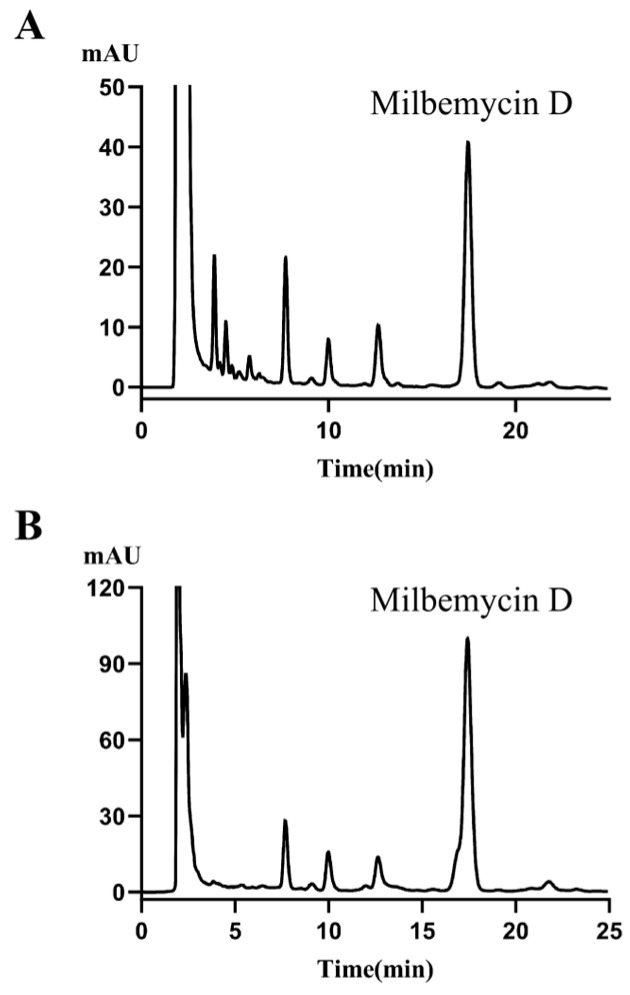
Screening for high-producing clones of *S. avermitilis* HU501-M. Representative HPLC chromatograms of fermentation broths from two conjugant clones: (**A**) clone 41 (typical titer) and (**B**) the high-producing clone 347, selected for further study.

To identify the highest-producing engineered clone, we screened the approximately 300 PCR-verified exconjugants (Figure 7A,B). Initial fermentations in the original medium revealed that most clones produced milbemycin D at titers between 120 and 150 mg/L. One clone, strain 347, consistently exhibited a significantly higher yield of 377.4 mg/L (Figure 7B). This clone was re-isolated as a single colony, and its genotype was reconfirmed by PCR (Figure 5B, lane 2) before being selected as the lead strain for all downstream optimization (Figure 7A shows a representative lower-producing clone for comparison).

### 3.5. Fermentation Process Optimization for High-Yield Milbemycin D Production

**Figure 8:**
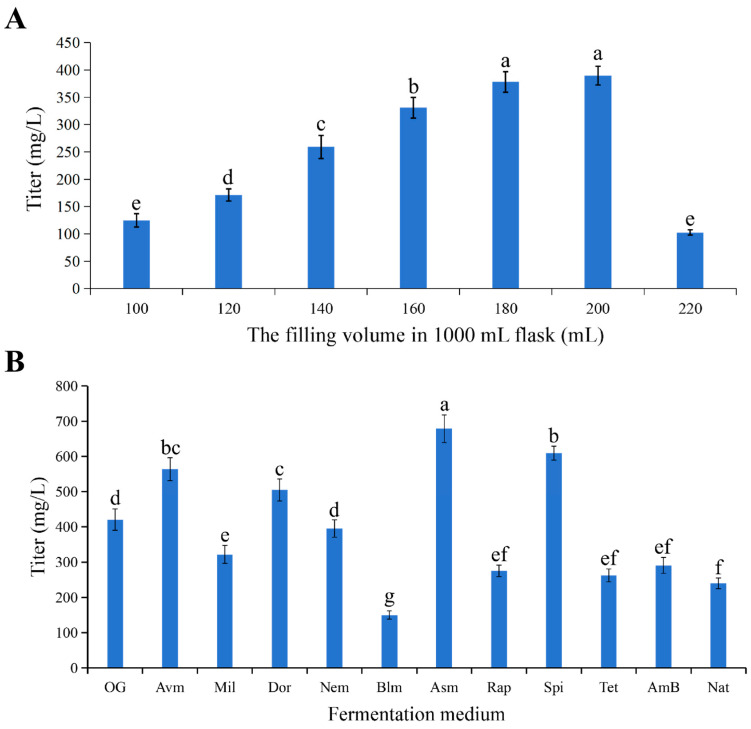
Fermentation optimization for milbemycin D production in *S. avermitilis* HU501-M strain 347. (**A**) Optimization of culture volume. Effect of medium fill volume in 1000 mL flasks on the final titers of milbemycin D. (**B**) Screening of culture media. Milbemycin D titers in 12 different media formulations developed for macrolide production. OG, original fermentation medium; Avm, avermectin production medium; Mil, milbemycin production medium; Dor, doramectin production medium; Nem, nemadectin production medium; Blm, bleomycin production medium; Asm, ascomycin production medium; Rap, rapamycin production medium; Spi, spiramycin production medium; Tet, tetramycin production medium; AmB, amphotericin B production medium; Nat, natamycin production medium. Data represent mean ± SD (*n* = 4). Different letters on the column represent significant difference at *p* < 0.05 probability level. The comparison was conducted on one-way analysis of variance.

With the high-producing clone *S. avermitilis* HU501-M strain 347 identified, we next optimized key fermentation parameters to maximize milbemycin D yield. Initial attempts to directly scale the culture from 30 mL in 250 mL flasks to 250 mL in 1000 mL flasks failed, suggesting oxygen transfer limitations. Dissolved oxygen (DO) was not measured directly during these shake-flask experiments; however, the oxygen limitation hypothesis was supported by subsequent bioreactor studies, which confirmed that product formation required DO levels above 80%, while failed fermentations corresponded with DO below 20%. A systematic evaluation of fill volume revealed that 200 mL was optimal in a 1000 mL flask; titers declined sharply with larger volumes (Figure 8A). This indicates that exceeding 200 mL likely causes oxygen depletion due to excessive biomass, thereby inhibiting secondary metabolism. Mycelial morphology was not systematically assessed during this optimization, representing a point for future investigation.

Subsequently, we evaluated 12 different culture media originally designed for the production of various macrolide antibiotics. The ascomycin production medium (Asm) supported the highest milbemycin D titer. Under these optimized conditions (200 mL fill volume in Asm medium), strain 347 achieved a final production potency of 679.03 mg/L (Figure 8B). This represents a 1.62-fold increase over the titer obtained in the original fermentation medium and establishes a robust process for the efficient biosynthesis of milbemycin D.

### 3.6. Enhanced Bioactivity of Biosynthesized Milbemycin D

We next evaluated the biological activity of the biosynthesized milbemycin D against three economically important pests: the pinewood nematode (*Bursaphelenchus xylophilus*), the fall webworm (*Hyphantria cunea*), and the diamondback moth (*Plutella xylostella*). Its efficacy was compared directly to the commercially relevant analogs, milbemycin A3 and A4.

Bioassays confirmed a dose-dependent lethal effect for all three compounds. Notably, milbemycin D consistently exhibited superior potency (Table 2). Against *B. xylophilus* and *H. cunea*, the LC_50_ of milbemycin D was 12.42 mg/L and 14.56 mg/L, respectively, which was 18.77% and 10.78% lower (i.e., more potent) than milbemycin A3, and 12.35% and 7.5% lower than milbemycin A4. The most pronounced enhancement was observed against *P. xylostella*, where milbemycin D (LC_50_ = 0.31 mg/L) was approximately 18–24% more potent than the A3 and A4 analogs. Based on non-overlapping 95% confidence intervals (Table 2), these differences were statistically significant.

## 4. Discussion

The milbemycins, produced by various *Streptomyces* species, represent a critical class of 16-membered macrolides with potent anthelmintic and insecticidal properties [47,48,49,50]. Their safety profile, stemming from poor blood–brain barrier permeability in mammals and the selective targeting of invertebrate glutamate-gated chloride channels and GABA receptors, has cemented their status as environmentally benign pest control agents [4,51,52]. This has led to their widespread registration and use across dozens of countries for protecting high-value crops [53,54]. The commercial product milbemectin, a mixture of milbemycin A3 and A4, exemplifies the success of this family [1]. However, the congener milbemycin D has long been recognized for its superior and more persistent efficacy against parasites like *Dirofilaria immitis* [7,55]. Despite this promise, its development has been stalled by its status as a low-yield fermentation byproduct, leading to prohibitive production costs and its eventual replacement by semi-synthetic alternatives like milbemycin oxime [56,57]. Our study directly addresses this decades-old bottleneck. By employing combinatorial biosynthesis driven by an optimized CRISPR/Cas9 system, we engineered *S. avermitilis* HU501-M, a strain where milbemycin D is no longer a trace component but the predominant product (Figure 6). Achieving a titer of 679.03 mg/L (Figure 8B) and confirming enhanced bioactivity (Table 2), this work transitions milbemycin D from a scientific curiosity to a development-ready agrochemical lead.

The success of this strategy hinges on precise biosynthetic logic. The structural divergence between milbemycin A3/A4 and milbemycin D originates at the first step of polyketide chain assembly, governed by the starter unit selectivity of the loading acyltransferase (AT) domain. While A3/A4 incorporates acetyl-CoA or propionyl-CoA, milbemycin D requires isobutyryl-CoA [11,58,59]. Rather than re-engineering the AT specificity in a native milbemycin producer—a complex endeavor—we adopted a strategic chassis-based approach. We utilized *S. avermitilis* HU501, a strain derived from the engineered 25-methyl and 25-ethyl ivermectin producer AVE-H39 [19] through classical mutagenesis. This two-step approach provided an ideal chassis: the AVE-H39 background contributed the pre-integrated enoyl reductase (ER) domain responsible for C22–C23 saturation, while the subsequent mutagenesis and screening for HU501 yielded a strain with a high-flux pathway already primed for isobutyryl-CoA incorporation (as evidenced by its production of ivermectin B1b). This effectively bypassed the two primary metabolic engineering challenges associated with producing milbemycin D in a heterologous host. The remaining engineering goal was to eliminate the C13 oleandrose disaccharide. The key modification was the replacement of the entire *aveA3* PKS module with *milA3* (Figure 3). This swap alters the reductive logic at the corresponding stage of chain elongation, converting the C13 carbonyl to a methylene group and thereby blocking the glycosylation cascade catalyzed by downstream glycosyltransferases [60]. Our results validate that whole-gene PKS replacement is an effective strategy for functional pathway refactoring. Attempts at finer-scale, inter-PKS domain swaps, such as introducing rapamycin modules into the avermectin PKS, have historically suffered from poor compatibility and low yield, underscoring the practical advantage of swapping entire, evolutionarily matched catalytic units [61].

A key methodological advance in this work is the application of a finely tuned CRISPR/Cas9-AcrIIA4 system for large-scale, seamless PKS replacement in *Streptomyces* [32]. Traditional genetic manipulation in this genus, reliant on suicide vectors and inefficient double-crossover recombination, is notoriously slow and labor-intensive, creating a major barrier for combinatorial biosynthesis projects [20,62]. While recombineering systems like Cre-loxP and meganucleases offered improvements, they often leave genetic scars or require pre-engineered sites [21,22,23,24,25]. The CRISPR/Cas9 system, particularly when optimized to mitigate toxicity and off-target effects, has significantly advanced this field [29,30,31,32]. Previously, its application in *Streptomyces* was largely confined to gene knockouts and point mutations to deregulate or enhance existing pathways [63,64]. Here, we demonstrate its power for a more complex task: the precise, scarless substitution of a large (~17.4 kb), high-GC content PKS gene within a chromosomal biosynthetic cluster (Figure 4 and Figure 5). Our cloning strategy, combining CRISPR/Cas9 targeting with seamless ligation cloning extract (SLiCE) in vitro assembly, ensured accurate integration and streamlined mutant isolation [65]. This efficient workflow is a significant technical contribution, providing a replicable blueprint for refactoring other complex biosynthetic pathways.

The tangible outcomes of this engineering are twofold. First, we achieved a substantial and optimizable yield of milbemycin D. The titer of 679.03 mg/L, representing a 1.62-fold increase through medium optimization (Figure 8B), demonstrates the high production potential of this engineered strain at the laboratory scale. Second, and crucially, bioassays confirmed that the biosynthesized compound retains—and indeed exceeds—the predicted bioactivity. The lower LC_50_ values of milbemycin D against the pinewood nematode (*Bursaphelenchus xylophilus*), fall webworm (*Hyphantria cunea*), and diamondback moth (*Plutella xylostella*) compared to A3 and A4 standards (Table 2) validate the long-held hypothesis that its unique isopropyl group at C25 confers enhanced potency [56,66]. This establishes a clear structure–activity relationship and underscores the value of accessing this previously scarce congener.

A meaningful evaluation of the production improvement achieved in this study requires appropriate benchmarking. Direct comparison with the native milbemycin D producer, *S. bingchenggensis* BCW-1, is complicated by the fact that milbemycin D is produced only as a minor byproduct in that strain, with reported titers of approximately 1.0–1.3 mg/L, representing less than 0.01% of total fermentation output [67]. In contrast, the primary achievement of our engineering strategy is not merely an increase in absolute titer, but a fundamental redirection of metabolic flux. In the engineered HU501-M strain, milbemycin D constitutes 50–55% of the total fermentation products (and up to 66.7% in the high-producing strain 347). This dramatic shift in product profile is arguably more significant than the absolute titer increase, as it transforms milbemycin D from a difficult-to-purify trace component into the dominant fermentation product, thereby enabling economically viable downstream processing. The final titer of 679.03 mg/L, achieved with minimal optimization, establishes a strong foundation for future strain improvement through classical mutagenesis, further gene editing, and fermentation process development.

Despite this success, our study also highlights a persistent scientific gap: the unpredictable compatibility between heterologous PKS modules and the metabolic context of the chassis strain, which can limit the yield of desired products. Furthermore, the stability of such extensively engineered strains over long-term fermentation and the potential for metabolic burden remains to be fully characterized. Looking forward, the engineered *S. avermitilis* HU501-M strain serves as an excellent platform for further development. Future work should focus on systematic metabolic engineering—such as co-expressing regulatory genes like *milR* or precursor pathway genes—to push titers even higher [14]. Additionally, the CRISPR/Cas9-mediated PKS swapping platform established here invites the exploration of creating novel “unnatural” milbemycin/ivermectin hybrids by exchanging other PKS modules, potentially uncovering derivatives with novel bioactivity spectra or improved safety profiles. In conclusion, this work successfully bridges microbial genetics and agricultural chemistry, providing both a solution to a specific production challenge and a generalizable framework for the rational design of next-generation polyketide-derived agrochemicals.

## 5. Conclusions

In conclusion, we demonstrate that the high-value compound milbemycin D can be established as the primary fermentation product in an engineered *S. avermitilis* strain through CRISPR/Cas9-mediated replacement of the *aveA3* polyketide synthase module. This strategy successfully redirected biosynthesis from ivermectin B1b to milbemycin D, yielding a titer of 679.03 mg/L with enhanced bioactive potency. Our work validates a refined genome-editing platform for precise, large-scale DNA integration in *Streptomyces*, enabling efficient combinatorial biosynthesis. This work provides a foundational strategy for developing a promising agrochemical and offers a generalized framework for engineering novel polyketide derivatives. The engineered strain and methods reported here represent a critical first step; further research into fermentation scale-up, formulation, and field efficacy will be essential to translate this proof-of-concept into a viable commercial product.

## Figures and Tables

**Table 1:** Strains and plasmids used in this study.

Strains/Plasmids	Description	References
Strains		
*E. coli* DH5α	Host for plasmids construction and cloning	Weidi bio
*E. coli* ET12567/pUZ8002	Donor strain for conjugation	Weidi bio
*S. avermitilis* AVE-H39	The 25-methyl and 25-ethyl ivermectin-producing strain	Zhang et al. [19]
*S. bingchenggensis* BCW-1	The milbemycin-producing strain	Wang et al. [11]
*S. avermitilis* HU501	Mutant strain of *S. avermitilis* MA-4680 with ivermectin B1b-producing	This study
*S. avermitilis* HU501-M	Genome editing strain of *S. avermitilis* HU501 with *aveA3* replaced by *milA3*	This study
Plasmids		
pKC1139-Cas9	*E. coli*-*Streptomyces* shuttle vector with Cas9 protein sequence, Am^R^	Jiang et al. [32]
pKC1139-Cas9-spacer	pKC1139-Cas9-based plasmid containing sgRNA sequence from *aveA3*, Am^R^	This study
pKC1139-Cas9-spacer-AA3UD	pKC1139-Cas9-spacer-based plasmid containing *aveA3* upstream and downstream fragments, Am^R^	This study
pKC1139-Cas9-spacer-AA3UD-MA3part	pKC1139-Cas9-spacer-AA3UD-based plasmid containing part of *milA3*, Am^R^	This study
pKC1139-Cas9-spacer-AA3UD-MA3	pKC1139-Cas9-spacer-AA3UD-MA3part-based plasmid containing full length of *milA3*, Am^R^	This study

Note: Am^R^—apramycin resistance.

**Table 2:** Nematicidal and insecticidal activity of milbemycin D compared to milbemycin A3 and A4.

Sample	*B. xylophilus* LC_50_ (mg/L) (95% CI)	*H. cunea* LC_50_ (mg/L) (95% CI)	*P. xylostella* LC_50_ (mg/L) (95% CI)
Milbemycin D	12.42 (9.41–16.41)	14.56 (10.71–19.78)	0.31 (0.24–0.40)
Milbemycin A3	15.29 (11.50–20.32)	16.32 (11.94–22.32)	0.41 (0.32–0.53)
Milbemycin A4	14.17 (10.71–18.75)	15.74 (11.58–21.42)	0.38 (0.30–0.49)

Note: LC_50_ values and 95% confidence intervals (CI) were determined by probit analysis. Differences between compounds were assessed by comparing 95% CIs; non-overlapping intervals indicate statistically significant differences (*p* < 0.05). Based on this criterion, milbemycin D was significantly more potent than both milbemycin A3 and A4 against all three target pests.

## Data Availability

All data generated or analyzed during this study are included in this manuscript and its Supplementary Materials. Requests for any additional information can be made to the corresponding author.

## Supplementary Materials

The following supporting information can be downloaded at: https://www.mdpi.com/article/10.3390/biology15070535/s1, Figure S1: HPLC purity analysis of purified milbemycin D used for bioassays. Detection at 240 nm. Purity: 97.715% by peak area integration. Figure S2: The original electrophoretogram includes the marker ladder, sample name, date, and condition. Figure S3: The ESI-MS spectra of milbemycin D; Figure S4: ^1^H NMR spectrum of milbemycin D in CDCl_3_ (400 MHz); Figure S5: ^13^C NMR spectrum of milbemycin D in CDCl_3_ (100 MHz); Figure S6: DEPT135 NMR spectrum of milbemycin D in CDCl_3_ (100 MHz); Table S1: Primers used for gene cloning, constructing and confirming the mu-tant; Table S2: Comparison of fermentation products in parental and engineered strains; Table S3: The NMR data of milbemycin D (in CDCl_3_, 400 MHz).
