# Supplementary material for: CRISPR/Cas9-Mediated Polyketide Synthase Replacement for High-Yield Biosynthesis and Biological Activity of Milbemycin D
Source: Biology (Basel). 2026 Mar 27;15(7):535. doi: 10.3390/biology15070535 (PMC13071997; doi:10.3390/biology15070535)
Supplement: Supplementary file 1 [file biology-15-00535-s001.zip › biology-4160079-supplementary.pdf]

Milbemycin D | DAD1A, Sig–240, 4 Ref–off

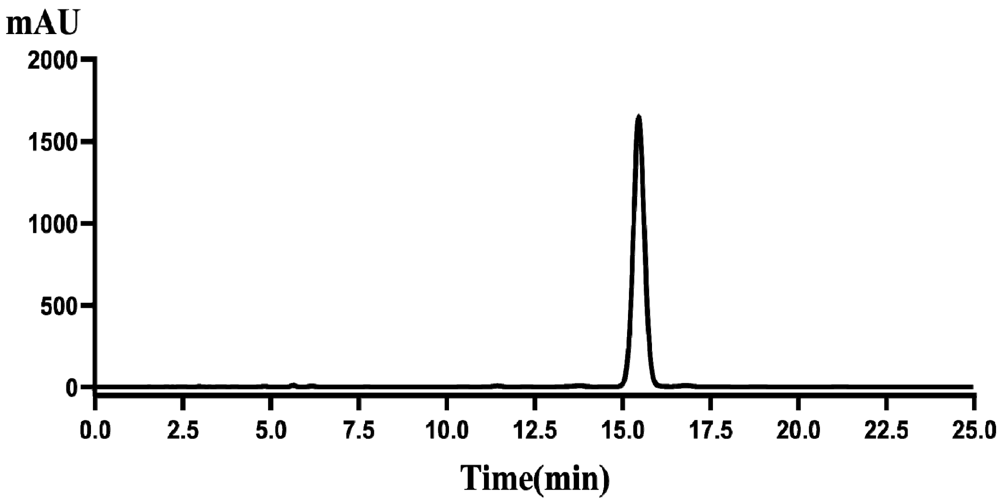

Peak #	RT(min)	Peak Area	Peak Area (%)	Peak Height	Start Time (min)	End Time (min)
1	1.559	8.194	0.021	2.042	1.299	1.671
2	2.972	20.776	0.054	2.513	2.812	3.172
3	4.825	42.131	0.110	3.749	4.405	5.292
4	5.637	87.871	0.230	9.208	5.312	5.904
5	6.170	58.155	0.152	5.165	5.905	6.542
6	10.473	30.493	0.080	1.892	10.079	10.795
7	11.455	110.225	0.288	5.717	10.799	11.945
8	13.786	189.796	0.496	7.054	12.792	14.199
9	15.461	37393.238	97.715	1644.243	14.705	16.347
10	16.798	217.670	0.569	7.938	16.347	17.295
11	18.892	45.661	0.119	1.702	18.272	19.412
12	21.195	63.604	0.166	2.167	20.599	21.865

Figure S1. HPLC purity analysis of purified milbemycin D used for bioassays. Detection at 240 nm. Purity: 97.715% by peak area integration.

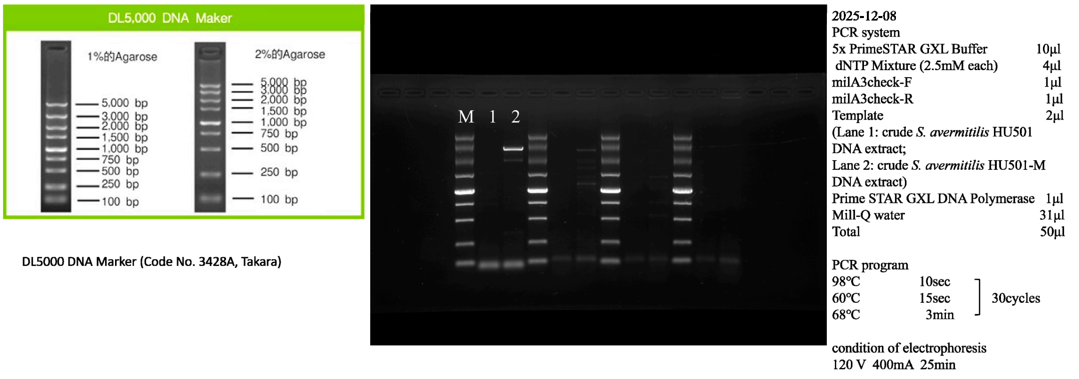

PCR verification using primers milA3check-F/R. Genomic DNA templates: Lane M, DL 5000 Marker (Code No. 3428A, Takara); Lane 1, wild-type S. avermitilis HU501; Lane 2, engineered S. avermitilis HU501-M. The expected 3.1-kb milA3 fragment is present only in the engineered strain.

Figure S2. The original electrophoretogram includes the marker ladder, sample name, date, and condition.

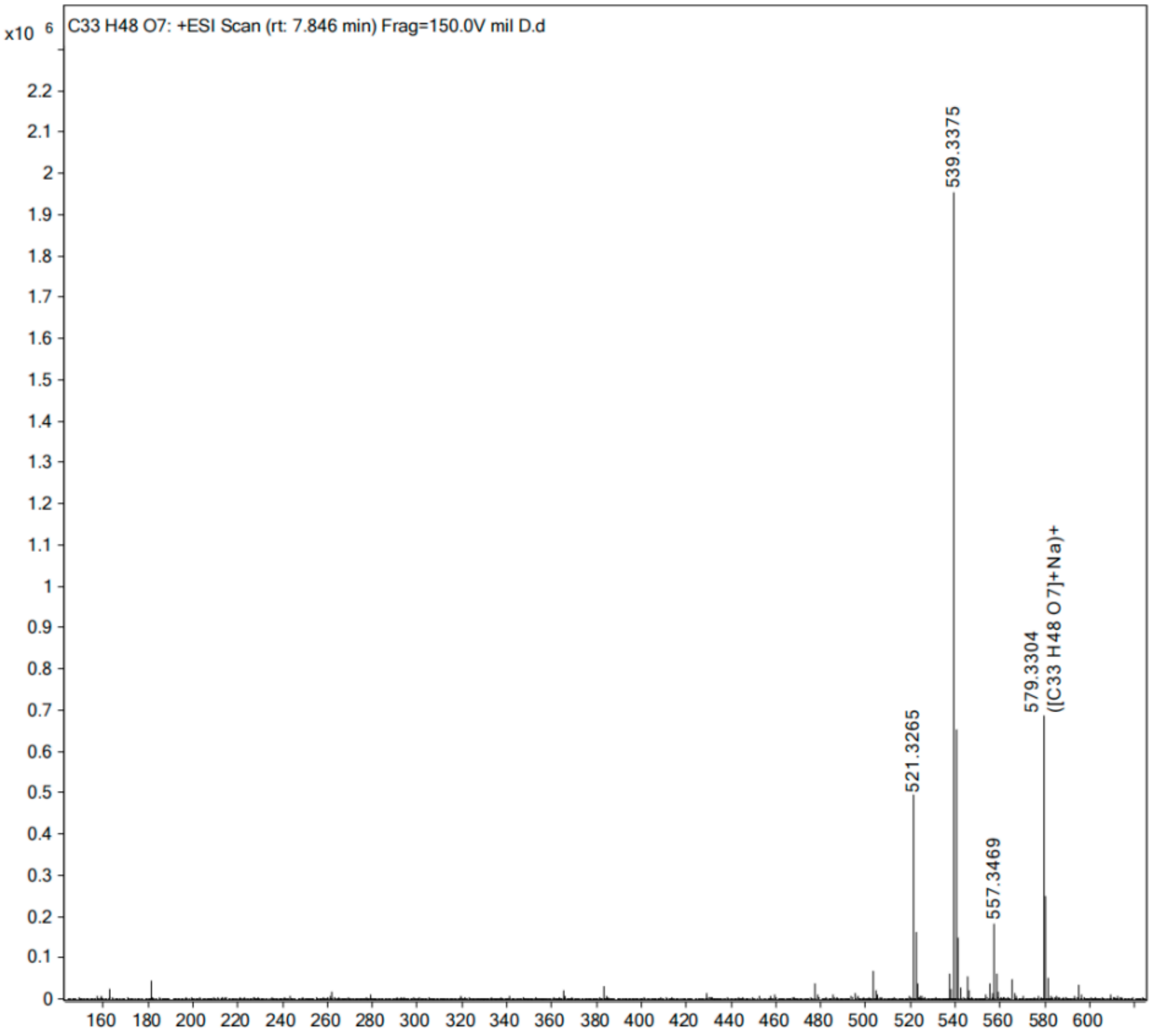

Figure S3. The ESI-MS spectra of milbemycin D

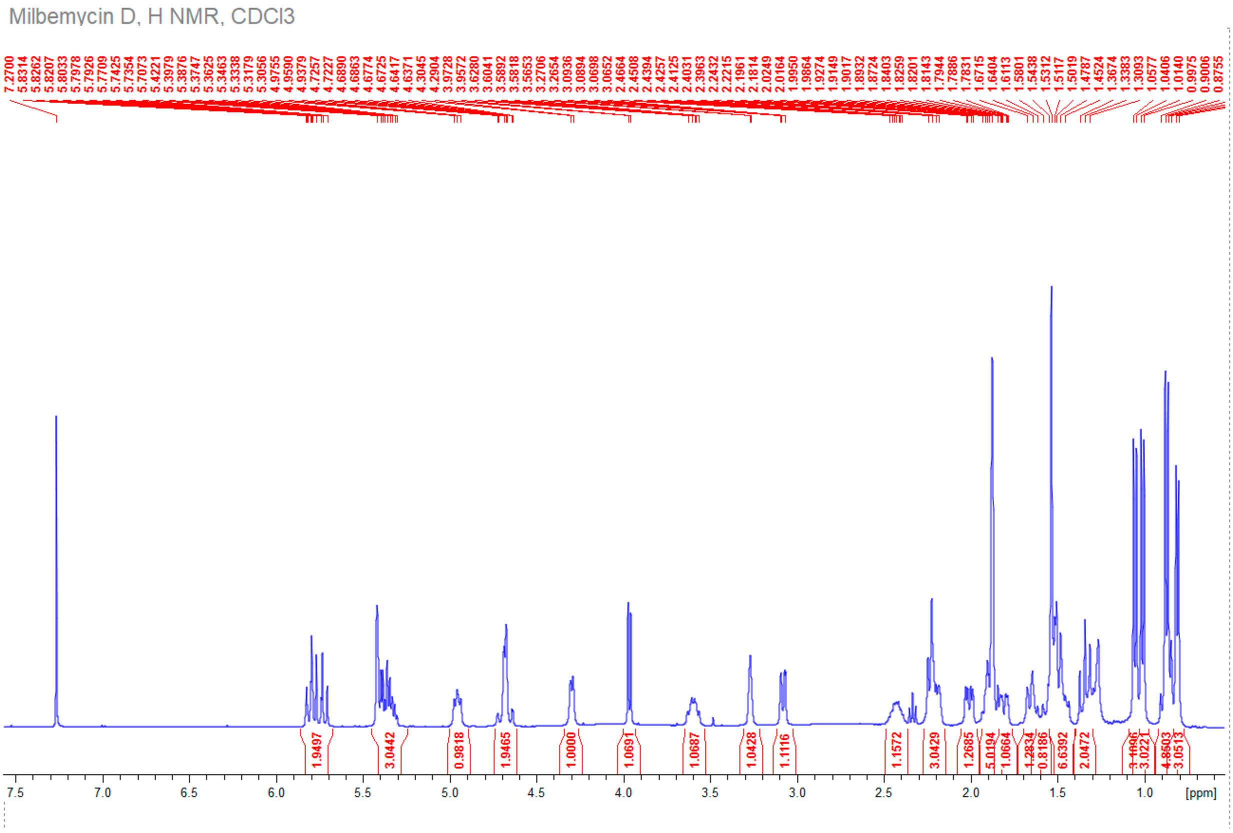

Figure S4. ^{1}H NMR spectrum of milbemycin D in $CDCl_3$ (400MHz)

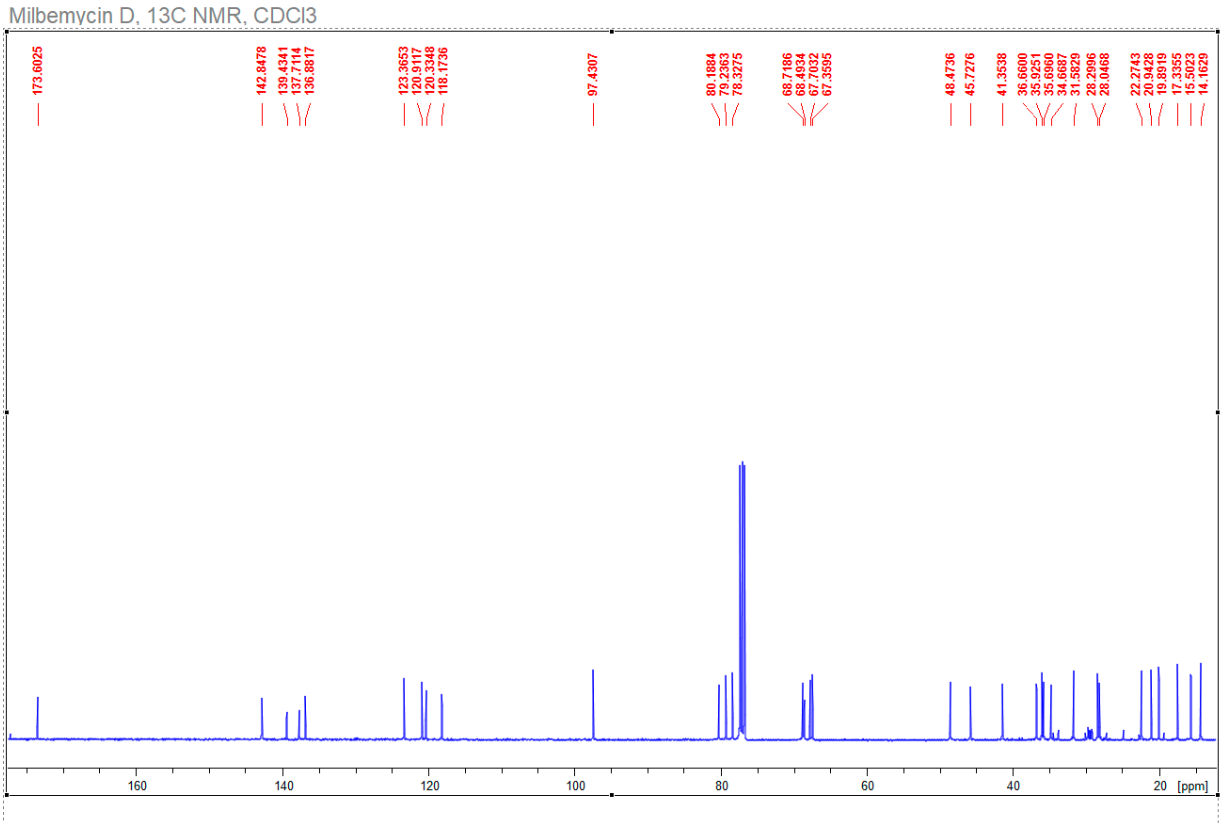

Figure S5. ^{13}C NMR spectrum of milbemycin D in $CDCl_3$ (100MHz)

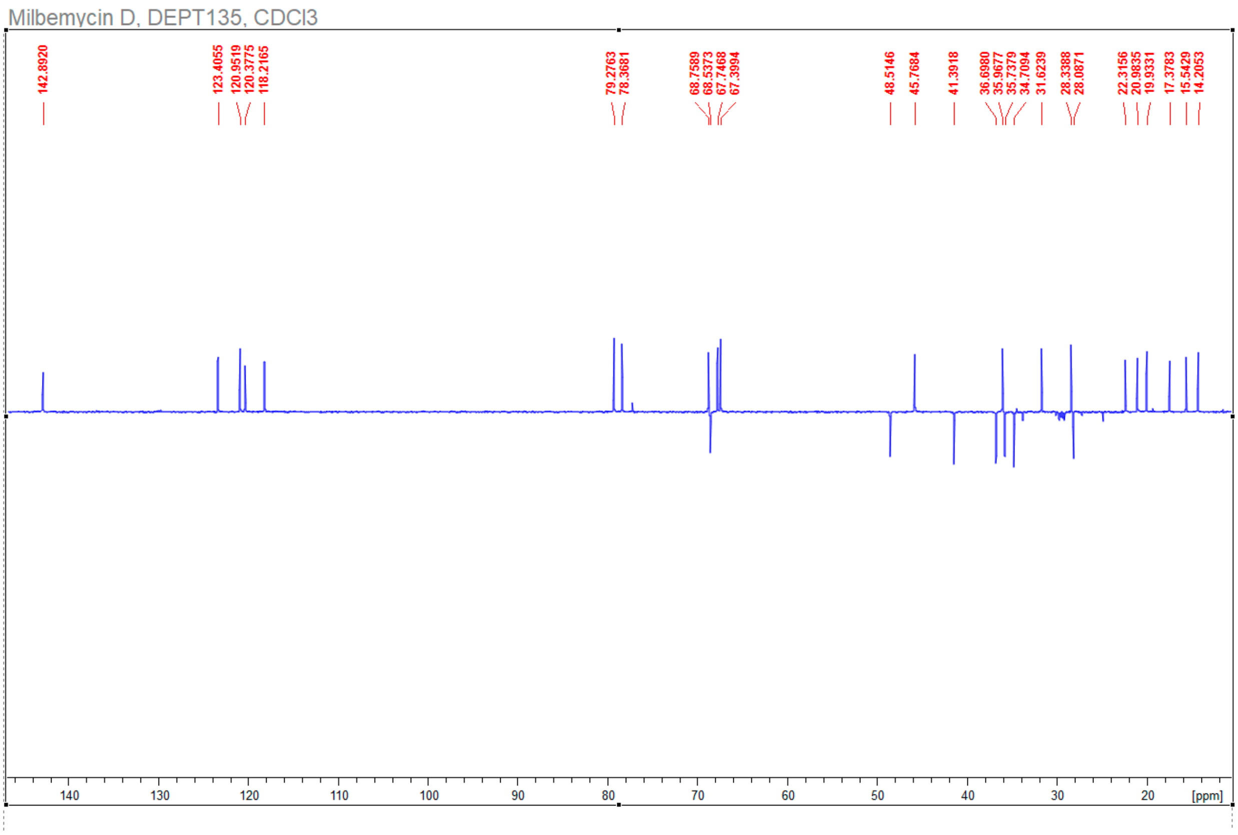

Figure S6. DEPT135 NMR spectrum of milbemycin D in $CDCl_3$ (100MHz)

Table S1: Primers used for gene cloning, constructing and confirming the mutant

primer	sequence
T1	GCTATTTCTAGCTCTAAAACggattgttgcgtgtgatgtgGCTGGATCCTACCAACCG
T2	GCGGCGACCACCACCACCAC
T3	TGAGCGGATAACAATTCCCC
T4	GTTTTAGAGCTAGAAATAGC
T5	CTCTTGATCCCCATCGAATTCcgaaaccggacacaccaca
T6	ttgaatttgggagacagtgCATATGagcccagaaaccactccga
T7	cactgtctcccaaattcaagaagtc
T8	ACAGCTATGACATGATTACtaccacgaccacctcttccg
milA3check-F	ccctcggtctgctcccaaactccccag
milA3check-R	ctggacccacacctcgaccatcccctg

Note: Capital letter: The sequence from pKC1139-Cas9; Small letter: The sequence from genomic DNA of *S. avermitilis* or *S. bingchenggensis*. Red letter: sgRNA (spacer); Blue letter: Restriction site.

Table S2. Comparison of fermentation products in parental and engineered strains.

Strain	Major Product(s)	Milbemycin D Titer (mg/L)	Milbemycin D (% of total)
S. Avermitilis AVE-H39 [19]	25-methyl and 25-ethyl ivermectin	Not detected	N/A
S. Avermitilis HU501	Ivermectin B1b	Not detected	N/A
S. Avermitilis HU501-M (strain 41)	Milbemycin D + minor components	~130-150	~50-55%
S. Avermitilis HU501-M (strain 347)	Milbemycin D	377.4 (initial), 679.03 (optimized)	~66.7%
S. Bingchenggensis BCW-1 [11]	Milbemycins A3/A4 (major), D (trace)	~1.0-1.3 (reported) [68]	~0.005%

Note: The C22-C23 saturation in HU501 and its derivatives is inherited from the pre-engineered enoyl reductase (ER) domain present in the parental strain AVE-H39, not from the subsequent random mutagenesis.

Table S3: The NMR data of milbemycin D(in $CDCl_3$,400 MHz).

No.	δ_H (*J* in Hz)	δ_C (p.p.m)
1		173.6
2	3.27 (m)	45.7
3	5.42 (m)	118.2
4		137.73
5	4.30 (d, 5.6)	67.7
6	3.96 (d, 6.2)	79.2
7		80.2
8		139.4
9	5.81 (dt, 11.2, 2.1)	120.3
10	5.74 (dd,14.2, 11.2)	123.4
11	5.39 (dd,14.2, 10.0)	142.8
12	2.43 (m)	35.9
13	2.20 (m)	48.5
	1.87 (m)	
14		136.9
15	4.96 (t, 8.4)	120.9
16	2.22 (m)	34.7
17	3.60 (m)	67.4
18	1.80 (m)	36.7
	0.87 (m)	
19	5.35 (m)	68.7
20	2.01 (m)	41.4
	1.34 (t, 11.6)	
21		97.4
22	1.66 (m)	35.7
	1.49 (m)	
23	1.51 (m)	28.0
24	1.48 (m)	31.6

25	3.08 (dd, 9.7, 1,8)	78.3
26	1.87 (br s)	19.9
27	4.71 (dd, 14.2, 2.1)	68.5
	4.66 (dd, 14.2, 2.1)	
28	1.01 (d, 6.6)	22.3
29	1.53 (br s)	15.5
30	0.81 (d, 6.1)	17.3
31	1.89 (m)	28.3
32	0.87 (d, 6.8)	14.2
33	1.05 (d, 6.8)	20.9
